# A Deep Convolutional Neural Network Model for Lung Disease Detection Using Chest X-Ray Imaging

**DOI:** 10.1155/pm/6614016

**Published:** 2025-06-24

**Authors:** Samia Dardouri

**Affiliations:** ^1^Department of Computer Science, College of Computing and Information Technology, Shaqra University, Shaqra, Saudi Arabia; ^2^InnoV'COM Laboratory-Sup'Com, University of Carthage, Tunis, Tunisia

**Keywords:** Adam optimizer, CNN, deep learning, feature extraction, image data augmentation, lung disease detection

## Abstract

Lung diseases, including pneumonia and COVID-19, are prevalent globally, necessitating early diagnosis for effective treatment. Medical imaging is widely regarded as an effective method for detecting lung diseases. Numerous researchers have dedicated their efforts to developing advanced detection techniques, significantly contributing to the prevention and management of these conditions. Despite advancements in imaging diagnostic methods, chest radiographs remain pivotal due to their cost-effectiveness and rapid results. This study proposes an automated system for detecting multiple lung diseases in x-ray and CT scans using a customized convolutional neural network (CNN) alongside pretrained models and an image enhancement approach. The dataset used comprises 6400 images sourced from Kaggle, categorized into three classes: pneumonia, COVID-19, and normal. To address dataset imbalance, data augmentation techniques were applied. The model includes preprocessing and classification stages, achieving high performance metrics: 96% precision, 95.33% recall, 95.66% *F*1-score, and 97.24% accuracy, highlighting its effectiveness compared to other deep learning models.

## 1. Introduction

The lungs are vital organs responsible for respiration, facilitating the intake of oxygen and the removal of carbon dioxide during inhalation. This critical function underscores their essential role in human life and overall well-being. Lung diseases, such as tuberculosis, pneumonia, and COVID-19, significantly impact global health and require substantial funding for effective intervention strategies. The World Health Organization (WHO) estimates that billions of dollars will be allocated to combat these diseases between 2021 and 2023, highlighting their importance in public health initiatives. This funding is crucial for improving diagnosis, treatment, and prevention efforts worldwide [1].

Pneumonia, a severe lung infection caused by acute respiratory pathogens, typically results from inflammation in the lung sacs due to bacteria or viruses. As a leading global cause of mortality, pneumonia disproportionately affects vulnerable populations, including children under five (accounting for 18% of their deaths), the elderly, and individuals with underlying health conditions. Distinguishing between bacterial and viral pneumonia is essential for appropriate treatment, despite their similar initial symptoms. Early detection of pneumonia is critical for timely intervention to prevent complications and improve patient outcomes. While the urgency of diagnosing COVID-19 may subside following the pandemic, the diagnosis of pneumonia, particularly distinguishing between bacterial and viral pneumonia, remains a crucial and ongoing issue. Accurately distinguishing between bacterial and viral pneumonia is essential for making informed treatment decisions, as antibiotic treatment for bacterial pneumonia differs from the management of viral pneumonia, with overuse of antibiotics contributing to the growing issue of antibiotic resistance.

Furthermore, implementing efficient diagnostic systems is vital for the early detection of lung diseases [2].

Such systems enhance diagnostic accuracy, facilitating timely treatment and improved patient outcomes. Advanced technologies like machine learning (ML) and deep learning (DL) can significantly aid in analyzing medical imaging data, leading to better identification of conditions such as pneumonia, tuberculosis, and COVID-19. While numerous imaging diagnostic methods exist, many are prohibitively expensive and inaccessible to large segments of the population, particularly in low-resource regions. The shortage of radiology experts in these areas and long waiting times for diagnoses exacerbate disease severity and contribute to increased mortality rates. Although diagnostic radiography is cost-effective and rapid, it may lead to misinterpretations due to visualized opacities. In recent years, the use of ML and DL techniques in medicine has advanced significantly, particularly for detecting and diagnosing diseases through radiological images [3, 4]. The detection of lung diseases has notably benefited from these technological improvements, enhancing accuracy and efficiency in identifying various conditions from imaging data. This progress highlights the transformative impact of artificial intelligence on medical diagnostics. Utilizing x-ray and computed tomography (CT) scans enables early and accurate detection of various lung diseases, including pneumonia and COVID-19. These imaging techniques provide detailed insights into lung health, facilitating timely diagnosis and treatment interventions that can significantly improve patient outcomes.

To address existing challenges, recent studies have explored ML techniques specifically DL models like convolutional neural networks (CNNs) to aid in pneumonia diagnosis using high-resolution imaging modalities such as CT scans. One study developed a DL architecture tailored for diagnosing severe pneumonia cases from chest x-rays (CXRs) by leveraging a dataset from the Radiological Society of North America; it focused on specialized zones within CXRs to enhance diagnostic accuracy [5, 6]. DL, a subset of ML inspired by the brain's structure and function, has emerged as a powerful tool in medical image analysis. These algorithms excel at quantifying, identifying, and classifying patterns within medical images by learning features directly from data eliminating the need for manual feature design based on domain-specific knowledge. CNNs are a prominent example of DL models utilized in this context; they specialize in processing images and extracting low-level features while efficiently capturing temporal and spatial dependencies through filters. Unlike traditional feed-forward layers, CNNs significantly reduce computational complexity by sharing weights and utilizing fewer parameters. Consequently, CNNs offer an effective approach for medical practitioners to diagnose and classify specific medical conditions with accuracy [7, 8].

This paper is organized as follows: Section 2 presents related works that highlight current advancements in lung disease diagnosis using ML techniques.  Section 3 describes the datasets used in this study for training and evaluation purposes.  Section 4 outlines the proposed methodology employed to enhance diagnostic accuracy through DL models.  Section 5 discusses the results obtained from our experiments and their implications for clinical practice. Finally, Section 6 concludes the paper with a summary of findings and suggestions for future research directions.

## 2. Related Works

In the field of disease detection, numerous researchers have been actively engaged in developing automated detection models. DL techniques have emerged as valuable tools for enhancing productivity, especially in computer-assisted diagnosis technologies, notably within medical imaging, image classification, and image restoration [9, 10]. The Sharma and Guleria [2] proposed a DL model comprising several stages: data collection, preprocessing, feature extraction, training, testing, classification, and pneumonia prediction. During data preprocessing, the data is balanced and normalized within the range of 0–255. This normalized data is then inputted into the VGG16 model for feature extraction, which involves extracting pertinent features from the images, facilitating the classification and prediction process. With its 16 layers encompassing input, convolution, pooling, dense, and output layers, VGG16 enables comprehensive feature extraction. Significant challenges in pneumonia detection include the large patient volume and the shortage of medical experts and supporting staff. As a result, the development of DL-based methods for early pneumonia detection has garnered significant attention due to their potential to improve diagnostic accuracy and efficiency [10–15].

In [16], Lamia and Fawaz emphasized the rapid spread of pneumonia, a disease posing significant risks to individuals' health and well-being. Biomedical diagnosis of pneumonia typically involves various diagnostic tools and clinical feature assessments. However, limitations in expert availability and tool accessibility hinder these efforts. To address these challenges, the researchers developed a mobile application employing DL techniques to classify pneumonia cases. This prototype mobile app, utilizing neural networks, is accessible through high-level tools like Create ML, which simplifies the process by eliminating complexities such as determining neural network layers and initializing parameters. The dataset used included over 5000 real images, which were processed to train an image classification model. The approach is aimed at making such models more accessible to nonexperts.

In [17], data augmentation techniques were utilized to optimize a model, leading to slightly improved precision compared to the original version. The enhanced model was employed to develop a web application that processes images and provides predictions. This classification model achieved a prediction accuracy of 78%, and further parameter tuning (e.g., adjusting the number of epochs) was suggested for improvement. The study highlighted the importance of artificial intelligence in aiding healthcare professionals in early pneumonia detection.

The DL model in [18] focused on detecting pneumonia from CXR images, demonstrating that an increasing number of neural network layers does not consistently improve accuracy. Instead, an optimal number of layers was determined to achieve the highest accuracy.

In [19], a Deep CNN was proposed to detect pneumonia infections from CXRs. This CNN model was trained on a dataset of 12,000 images, with data augmentation employed to expand the dataset. The VGG19 network was used in this model, achieving a classification accuracy of 99.34%.

Several studies have addressed class imbalance issues, such as employing generative adversarial networks (GANs), including deep convolutional GANs (DCGAN) and Wasserstein GANs with gradient penalty (WGAN-GP), alongside random undersampling (RUS) techniques [20, 21]. The study in [22] optimized a DL model for detecting multiple lung diseases from CXRs, focusing on conditions like pneumonia, tuberculosis, and COVID-19, and enhancing diagnostic precision while reducing computational costs.

Similarly, [23] highlighted the contribution of DL models, particularly CNNs, in diagnosing lung diseases, integrating optimization techniques to enhance diagnostic performance. Al-Qaness et al. [24] provided a comprehensive survey of DL techniques in lung disease detection, exploring advancements, challenges, and future directions. Additionally, studies [25–32] presented various CNN-based models and ensemble approaches for classifying and segmenting lung diseases using CXRs and CT images, achieving high accuracy and demonstrating the potential of DL in this field.

This study proposes an efficient DL framework specifically designed for the detection of lung diseases using CXR images. The framework is aimed at achieving a balance between accuracy and complexity, offering a cost-effective solution for medical and radiology professionals. A large and diverse dataset is compiled from standard public repositories, including cases of healthy individuals, COVID-19, pneumonia, and normal conditions. The proposed approach employs data augmentation techniques to enhance the dataset's size and improve the model's generalization capabilities. By leveraging a CNN, the model acts as a feature extractor and classifier. The performance of the CNN and other DL models is explored and evaluated to ensure accurate classification of lung disease cases. In [33], a novel DL approach for detecting pneumonia from CXR images using a separable CNN. The method focuses on reducing the computational complexity and improving the efficiency of the model by employing separable convolutions. In the context of Generative AI techniques, specifically GANs, the studies in [34, 35] propose a framework that integrates GANs with traditional diagnostic methods to improve the quality and accuracy of lung disease detection, particularly for conditions like pneumonia and tuberculosis. This approach capitalizes on the capability of GANs to generate high-quality synthetic data, which can then be used to augment existing medical datasets, ultimately leading to improved model training. This is especially beneficial in cases where labeled data is either scarce or imbalanced, which is a common challenge in medical image analysis. By integrating GANs, the diagnostic process becomes more robust and precise, enabling the detection of subtle patterns in CXR images that may not be immediately noticeable to traditional diagnostic methods, thereby enhancing overall diagnostic performance. A significant stride in pneumonia detection was marked by Nasser, A.A., Akhloufi, M.A. [36], who harnessed the power of transfer learning and attention mechanisms in their DL approach for chest disease detection. The key contribution of this research is the integration of transfer learning, which leverages pretrained models on large datasets (like ImageNet) to improve performance with smaller, specialized datasets, such as CXR images. This technique helps in fine-tuning the model for the specific task of pneumonia detection, requiring fewer labeled images and reducing the need for extensive training from scratch [35, 36].  Table 1 gives an overview of the various models, methodologies, and contributions that have been made in the field of lung disease detection using CXR imaging, highlighting key performance indicators such as accuracy and method novelty.

## 3. Dataset Descriptions

**Figure 1:**
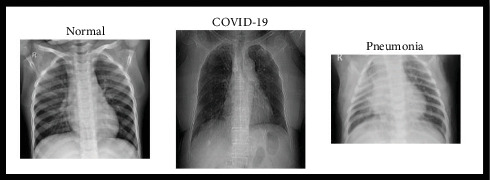
Samples of the dataset.

The Kaggle dataset [15] comprises a comprehensive collection of CXR images categorized into three distinct classes: COVID-19, pneumonia, and normal. This dataset is systematically organized into two main folders: train and test, each containing three subfolders corresponding to the aforementioned classes. In total, the dataset encompasses 6432 CXR images, with 20% allocated specifically for testing purposes. Prior to inclusion in the dataset, all chest radiographs underwent rigorous quality control screening to eliminate any low-quality or unreadable scans, ensuring the integrity of the data. Subsequently, the diagnoses for these images were meticulously assessed by two expert physicians to guarantee accuracy before being utilized for training the AI system. To further enhance the reliability of the dataset, a third expert verified the evaluation set to address any potential grading errors. This multitiered verification process significantly bolsters the dataset's credibility and usability in research and clinical applications.  Figure 1 illustrates sample images from the dataset, providing visual context for the classifications involved.

This robust dataset serves as a critical resource for developing and validating ML models aimed at improving diagnostic accuracy in lung disease detection. By leveraging high-quality, well-categorized images, researchers can train algorithms that not only enhance detection rates but also contribute to more effective treatment strategies for patients suffering from these serious respiratory conditions.

## 4. Proposed Methodology

The proposed DL framework for detecting lung diseases from CXR images is meticulously structured into three distinct phases:

• Data collection and preprocessing
• Feature extraction and model training
• Testing and evaluation

This comprehensive approach is aimed at enhancing diagnostic accuracy while ensuring the robustness and reproducibility of the model.

### 4.1. Data Collection and Preprocessing

The first phase involves several critical steps to prepare the dataset for effective analysis. A large and diverse dataset comprising 6432 CXR images was compiled from publicly available repositories, representing cases of normal (healthy) individuals, COVID-19, pneumonia, and other lung diseases. To ensure data quality and uniformity, preprocessing steps were applied, including image resizing to standardize dimensions, pixel normalization to scale intensity values for numerical stability, and data augmentation techniques such as rotation, flipping, zooming, and brightness adjustments. These augmentations effectively increased the number of training instances, particularly for underrepresented classes, enhancing the model's generalization and robustness.

### 4.2. Feature Extraction and Model Training

In the feature extraction phase, relevant features are derived from the preprocessed CXR images using advanced techniques, such as leveraging pretrained CNNs or custom-designed architectures. These extracted features are then used to train the DL model, where the architecture is optimized to classify cases accurately. Model training involves fine-tuning parameters, minimizing loss functions, and validating performance to ensure robust and reliable predictions.

### 4.3. Testing and Evaluation

The final phase involves assessing the model's performance through comprehensive evaluation metrics. The CNN model is trained using the augmented dataset, which provides a diverse set of examples to enhance learning. Performance evaluation includes metrics such as accuracy to measure overall correctness, precision to determine the proportion of correctly identified positive cases, and recall to assess the model's ability to detect all relevant instances. These metrics collectively ensure a thorough evaluation of the model's reliability in diagnosing lung diseases.

This comprehensive methodology not only enhances diagnostic accuracy but also provides a robust framework for future research endeavors in medical imaging and DL applications for lung disease detection. By integrating advanced computational techniques with innovative architectures, we aim to improve diagnostic performance significantly. To promote reproducibility in research, all source code and mathematical models will be made publicly available upon publication. This commitment ensures that other researchers can replicate our work effectively and build upon our findings in advancing lung disease detection methodologies.

In our study, we leveraged novel CNN model designs along with data augmentation to process our dataset, deploying three distinct architectures:

• Sequential model

This model is built by arranging layers in a strict, linear sequence where each layer's output becomes the input for the next. Such an arrangement enables the network to progressively learn more abstract features—an approach particularly effective for distinguishing infected from noninfected regions in CXRs. Our sequential model features five convolutional layers with an increasing number of filters as the network deepens. We set the activation's alpha parameter to 0.66 and employ Leaky ReLU so that even inactive neurons allow a small gradient (unlike standard ReLU, which cuts off the gradient completely). Furthermore, max pooling is applied after each activation to downsample the spatial dimensions, and the entire model is optimized using the Adam optimizer at a learning rate of 0.0001.

• Functional model

Offering greater flexibility than its sequential counterpart, the functional model permits arbitrary connections between layers rather than enforcing a simple one-after-the-other structure. This flexibility is particularly beneficial for constructing more complex architectures. In our implementation, the input first passes through an initial layer and then follows a custom pathway that includes two convolutional layers with 7 × 7 kernels. This is followed by an additional convolutional layer that applies a 1 × 1 kernel on top of a 3 × 3 kernel. The outputs from these layers are processed separately, concatenated, and then routed through five additional 3 × 3 convolutional layers. Training is performed from scratch with the Adam optimizer (learning rate = 0.0001), allowing the model to learn features tailored specifically to our task.

• Pretrained model

This approach relies on transfer learning, where a model with weights prelearned on large-scale datasets (VGG16) is used directly for classification. Rather than building a network from the ground up, the pretrained model capitalizes on the robust feature extraction capabilities developed during its original training. This method greatly reduces both training time and computational costs and is particularly effective when working with limited datasets.

**Figure 2:**
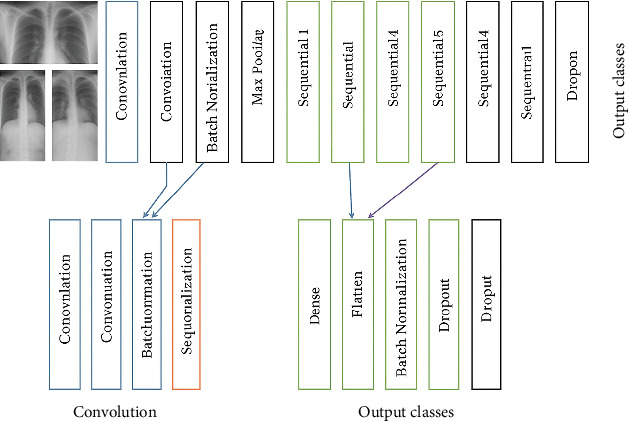
CNN structure [38].

The proposed DL framework has undergone multiple constructions and training sessions, exploring various parameters to select optimal hyperparameters and achieve a balanced performance architecture. Broadly, it comprises two primary stages. The initial stage involves several image preprocessing steps, including image resizing to obtain a standardized size and rescaling pixel values to fall within the [0, 1] interval. Subsequently, the second stage focuses on feature extraction and image classification using the proposed CNN models presented in Figure 2. The feature extraction stage constitutes the second component of the CNN architecture, comprising three blocks, each containing a convolution layer, maximum pooling layer, and dropout layer. Within the convolutional layer, input images are transformed into matrix representations. The convolution operation is applied between the input matrix and a feature kernel of a specified dimension, resulting in a feature map. This operation effectively reduces the dimensions of the image, facilitating further processing. Data augmentation methods [17, 39] prove beneficial in addressing the imbalance and scarcity of data in certain classes when dealing with limited and uneven datasets. This approach proves particularly useful for achieving a balance in the number of images across different MRI classes related to brain tumors and for expanding the overall dataset. Various augmentation techniques, including rotation, cropping, height and width adjustments, filling operations, zooming, and horizontal rotation brightening, are employed to augment images and rectify class imbalances. Given the unbalanced nature of our dataset, this augmentation technique is applied to artificially increase the number of images for each class, particularly those with fewer instances.

The images present in the dataset are of varying resolutions. However, CNN models require images to be of a specified uniform size. Therefore, all images in the dataset were resized to 224 × 224 pixels. Reducing the input image size facilitates faster processing and enables the model to perform the associated task more efficiently.

Data augmentation is a commonly used technique to significantly increase the volume of training data by introducing slight variations to an image during each training epoch. In this work, the augmentation variations include horizontal flip, zoom, shear, rotation, and rescaling. This technique is essential for achieving high accuracy, as it allows the CNN model to train on a larger and more diverse dataset than originally available.

Given the apparent class imbalance in the dataset, with potentially fewer instances of the “pneumonia” class compared to the “normal” class and the “COVID” class, a strategy to counter this issue involves leveraging data augmentation techniques. By employing data augmentation, we aim to augment the training dataset by generating synthetic examples, thereby increasing the number of instances available for training. This approach not only addresses class imbalance but also enhances the robustness and generalization ability of the ML model. To mitigate the risk of overfitting, expanding our dataset through artificial means is crucial. This involves introducing variations to the existing data via minor transformations, thereby increasing its size. Techniques that manipulate training data while preserving their labels are known as data augmentation methods. Common augmentations include grayscale conversion, horizontal and vertical flips, random cropping, color adjustments, translations, and rotations. By applying a subset of these transformations to our training data, we can substantially augment the number of examples, leading to the creation of a highly robust model. For data augmentation, we have opted to implement the following transformations:

• Randomly rotating some training images by up to 30°.
• Randomly zooming in or out on some training images by up to 20%.
• Randomly shifting images horizontally by 10% of their width.
• Randomly shifting images vertically by 10% of their height.
• Randomly flipping images horizontally.

**Figure 3:**
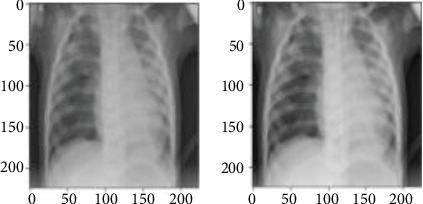
Augmentation data process: (a) original image; (b) augmented image.

Once our model is prepared, we proceed to fit the augmented training dataset shown in Figure 3.

**Figure 4:**
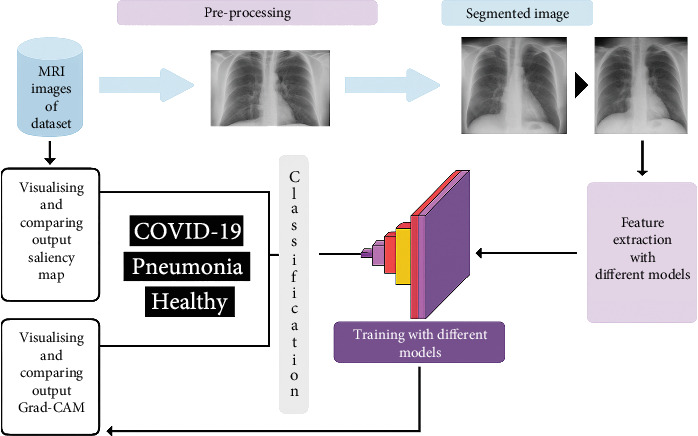
Proposed architecture for lung disease detection.

The proposed approach consists of two main steps. Firstly, we introduce a normalization method specifically designed for CXRs, with the goal of removing unnecessary components while retaining crucial information. Following this, Deep CNNs are utilized, with a preference for using the ADAM optimization function to build predictive models using the normalized dataset.  Figure 4 provides an overview of the proposed approach.

## 5. Results and Discussions

For assessing the performance of the model, several evaluation metrics are employed to gauge the classification outcomes, particularly for pneumonia classification from lung x-rays. The primary evaluation metrics utilized include accuracy, recall (sensitivity), and *F*1-score. These metrics are defined as follows:

• Accuracy: measures correct predictions. It is given by Equation (1):

(1) $$\begin{array}{c} Accuracy=\frac{TP+TN}{TP+TN+FP+FN}. \end{array}$$

**Recall** measures correctly identified positives. It is defined in Equation (2):

(2) $$\begin{array}{c} Recall=\frac{TP}{TP+FN}. \end{array}$$

**F**1**-score** balances precision and recall. It is calculated as shown in Equation (3)

(3) $$\begin{array}{c} F1-score=\frac{2}{\left(1/Precision\right)+\left(1/Recall\right)}. \end{array}$$

Here, TP denotes true positives, TN denotes true negatives, FP denotes false positives, and FN denotes false negatives. These metrics collectively provide a comprehensive evaluation of the model's performance in detection lung diseases cases from CXR images. These metrics together give a comprehensive view of model performance.

**Figure 5:**
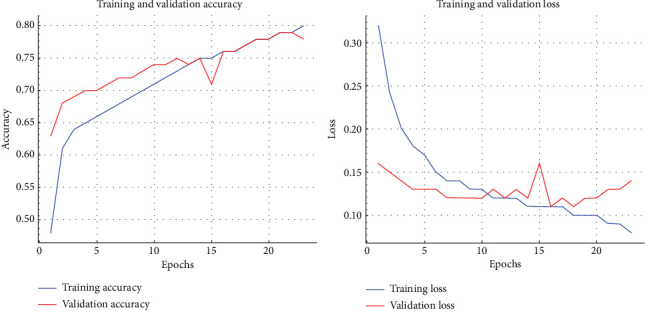
Model accuracy and loss over epochs.

The final output of the proposed method, using 20 epochs, is illustrated in Figure 5, which shows the model's accuracy and the corresponding loss with respect to the number of epochs. The model's accuracy increases significantly from the start, reaching approximately 0.97 by the third epoch and stabilizing at this level until the 20th epoch. Notably, training accuracy surpasses validation accuracy by the third epoch across all folds. The model is trained on four folds at each iteration, with performance metrics recorded and averaged to provide an overall estimate of the model's effectiveness.

**Figure 6:**
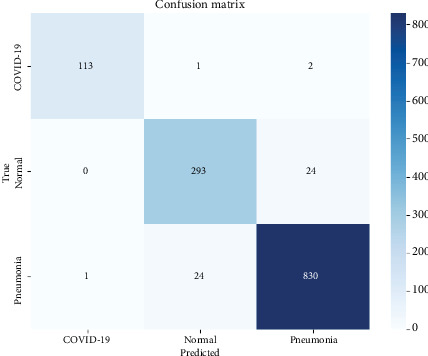
Confusion matrix.

The confusion matrix, presented in Figure 6, serves as a valuable tool for assessing which classes may be misclassified more frequently, as well as for overall model evaluation, monitoring, and management. It provides insights into the performance of the proposed system, aiding in identifying specific classes that may pose challenges for accurate classification. The neural network demonstrates remarkable performance, achieving an accuracy of 97.24%, as illustrated by the confusion matrix in Figure 6. Out of 1288 test images, only 52 were misclassified, primarily between the pneumonia, COVID-19, and normal classes. This suggests that while the model performs exceptionally well overall, distinguishing between these two conditions remains challenging due to subtle differences.

Our proposed model outperforms previously developed approaches, demonstrating an accuracy of 97.24%. Experimental results illustrate that the proposed CNN model exhibits superior convergence compared to other methods such as ANN approaches, random forest classifiers, transfer learning algorithms, and various CNN models. As indicated in Table 2, our model achieved an average recall of 95.33%, an average *F*1-score of 95.66%, and an average precision of 96%.

To compare the proposed model with existing techniques, the same dataset of 6432 lung disease images was utilized alongside various DL models and CNN architectures, including VGGNet and DenseNet121. Performance metrics for each technique are presented in Table 3, indicating that our model with embedded concatenate and transpose blocks outperforms other models in terms of accuracy and Dice score. This advancement offers hope for more accurate and early diagnoses, which can lead to better patient outcomes and a deeper understanding of disease progression.

Overall, these findings indicate that DL techniques can achieve high accuracy and performance in image analysis tasks related to lung disease detection. However, it is crucial to note that the specific architecture choice and the number of training images significantly impact performance outcomes. Therefore, selecting an appropriate architecture and optimizing hyperparameters tailored to the specific task and dataset is essential for achieving optimal results.

This comprehensive analysis underscores the effectiveness of our proposed methodology in enhancing diagnostic accuracy for lung diseases while providing a robust framework for future research endeavors in medical imaging and DL applications. Advanced models such as vision transformers (ViTs) [28] and EfficientNet [30] are designed for general-purpose image classification and may not be specifically optimized for pneumonia detection. Our custom CNN model is tailored for medical image analysis, incorporating domain-specific optimizations suited for CXR features. The use of fewer but highly optimized layers allows the model to focus on essential features, reducing computational complexity and overfitting risks. Additionally, extensive data augmentation techniques including rotation, flipping, brightness adjustments, and contrast normalization enhance generalization by increasing dataset diversity and mitigating sensitivity to imaging variations. Unlike deeper models that may suffer from overfitting on small datasets, our model balances computational efficiency and feature extraction by maintaining an optimal number of parameters while leveraging the Adam optimizer for dynamic learning rate adjustments.

Furthermore, our multibranch convolutional architecture captures both fine-grained and global patterns in x-ray images, enabling a richer representation of pneumonia-related abnormalities. This targeted approach ensures superior performance compared to standard single-stream CNNs and Transformer-based models, which rely on larger datasets. Achieving 97.24% accuracy, our model demonstrates that a well-optimized CNN, specifically designed for pneumonia detection, can match or even exceed the performance of more advanced architectures. Unlike prior studies, we validate our results using rigorous testing techniques to confirm that improvements are not merely due to dataset bias or overfitting. Ultimately, while ViT and EfficientNet are powerful for general image classification, our approach highlights that carefully designed, domain-specific architectures can outperform more complex but generic models in medical imaging tasks.

## 6. Conclusion and Future Work

The early detection of lung diseases is essential for timely diagnosis and effective treatment, significantly impacting patient outcomes. CNNs and other DL methods have demonstrated remarkable success in identifying areas affected by lung diseases. However, challenges such as variability in image quality, the presence of artifacts, and the similarities between COVID-19, pneumonia, and normal lung regions continue to pose significant hurdles. While ViT and EfficientNet are state of the art for general image classification, their computational complexity and reliance on extensive training data make them less suitable for specialized medical applications like pneumonia detection in CXRs. Our study demonstrates that carefully designed, domain-specific architectures when combined with effective data augmentation, efficient feature extraction, and adaptive optimization techniques can outperform more complex but generic models.

This study proposed a novel DL model that integrates convolution and concatenation blocks to enhance lung disease detection using CXR images. The automated method leverages DL for effective feature extraction from x-ray scans, aiming to achieve improved classification performance and faster learning rates compared to traditional models. Despite limitations imposed by a relatively small training dataset, experimental results demonstrate the effectiveness of the proposed model. Its success can be attributed to minimal preprocessing requirements and the elimination of handcrafted features, making it adaptable for various x-ray classifications.

Several avenues for future research can further enhance the capabilities of this proposed system. Validating the model beyond CXR images by incorporating other imaging modalities such as CT scans and magnetic resonance imaging (MRI) will help assess robustness and adaptability across different imaging techniques. Additionally, expanding the classification framework to include more labels for various lung diseases such as chronic obstructive pulmonary disease (COPD), lung cancer, and interstitial lung disease will provide a more comprehensive diagnostic tool for clinicians. Investigating explainable AI techniques will also be vital for enhancing trust in automated systems among healthcare professionals. Providing interpretable outputs that elucidate how decisions are made can facilitate better integration into clinical workflows. Finally, conducting longitudinal studies to evaluate the model's performance over time could provide insights into its effectiveness in tracking disease progression or response to treatment. By pursuing these directions, efforts are aimed at enhancing the accuracy, reliability, and applicability of this DL model in clinical practice, ultimately contributing to improved patient outcomes in lung disease management. This commitment reinforces the importance of advanced computational techniques in medical diagnostics and emphasizes the potential for innovation to benefit healthcare professionals and patients alike. In the realm of x-ray imaging, optimizing generative AI models and exploring innovative architectures like transformer-based models are promising directions to enhance scalability and adaptability. Additionally, focusing on x-ray-specific optimizations may further improve diagnostic accuracy. Finally, conducting extensive real-world clinical validations will be critical to demonstrating the reliability and applicability of these advanced systems in diverse and dynamic healthcare environments.

## Figures and Tables

**Table 1:** Overview of the various models in previous works.

**Study**	**Dataset**	**Method**	**Accuracy rate**
[2]	VGG16	Kaggle's pneumonia detection dataset	95.4%

[3]	YOLOv3	Curated dataset for COVID-19 posterior–anterior chest radiography images (x-rays).	92.15%

[4]	VGG16	Kaggle's pneumonia detection dataset	94%
	VGG19		95%
	Xception		96%

[5]	F-RNN-LSTM	Kaggle chest x-ray dataset	95.04%

[7]	PNET	X-ray images collected at the Shenzhen No.2 People's Hospital	92.79
	AlexNET		90.3
	VGG16		90.39

[8]	PneuNetV1	NIH chest x-ray dataset	91.23

[11]	DenseNet201	Kaggle chest x-ray dataset	97

[13]	EFA-Net	The NIH chest x-ray dataset	90.03%

[16]	CNN	The dataset of more than 5000 real images	84%

[37]	CNN	Kaggle chest x-ray dataset	94%

[23]	MDCXR3-Net	Kaggle chest x-ray dataset	97%

[25]	CNN	The dataset utilized in this research comprised 5400 CXR images	96%

[26]	LDC-BCBES	The dataset utilized in this research comprised 5400 CXR images	93.72%,
	DBN		82.47%,
	DQN		65.79%,
	RNN is		83.14%,
	LSTM		87.83%,
	SVM		87.81

[28]	Vision transformer (ViT)-based deep learning	Chest x-ray image dataset comprising 19,003 images	95.87%

[29]	CNN		95%

[30]	EfficientNetB0	Guangzhou Women and Children's Medical Center, Guangzhou.	95.19%

[31]	VDSNet model	The NIH chest x-ray dataset	73%

Proposed model	CNN + Adam optimizer	Kaggle chest x-ray dataset	97.24%

**Table 2:** Performance of proposed model.

**Technique**	**Precision**	**Recall**	**F**1**-score**
Bacterial pneumonia	0.97	0.97	0.97
Coronavirus disease (COVID-19)	0.99	0.97	0.98
Normal	0.92	0.92	0.92

**Table 3:** Comparative study.

**Study**	**Dataset**	**Accuracy rate**
[2]	VGG16	95.4%

[3]	YOLOv3	92.15%

[4]	VGG16	94%
	VGG19	95%
	Xception	96%

[5]	F-RNN-LSTM	95.04%

[7]	PNET	92.79
	AlexNET	90.3
	VGG16	90.39

[8]	PneuNetV1	91.23

[11]	DenseNet201	97

[13]	EFA-Net	90.03%

[16]	CNN	84%

[37]	CNN	94%

[23]	MDCXR3-Net	97%

[25]	CNN	96%

[26]	LDC-BCBES	93.72%,
	DBN	82.47%,
	DQN	65.79%,
	RNN is	83.14%,
	LSTM	87.83%,
	SVM	87.81

[28]	Vision transformer (ViT)-based deep learning	95.87%

[29]	CNN	95%

[30]	EfficientNetB0	95.19%

[31]	VDSNet model	73%

Proposed model	CNN + Adam optimizer	97.24%

## Data Availability

The datasets were collected from Kaggle, available at the following link: https://www.kaggle.com/datasets/prashant268/chest-xray-covid19-pneumonia.
